# Improvement of Inflammation and Pain after Three Months’ Exclusion Diet in Rheumatoid Arthritis Patients

**DOI:** 10.3390/nu13103535

**Published:** 2021-10-09

**Authors:** Maria Teresa Guagnano, Chiara D’Angelo, Daniela Caniglia, Pamela Di Giovanni, Eleonora Celletti, Emanuela Sabatini, Lorenza Speranza, Marco Bucci, Francesco Cipollone, Roberto Paganelli

**Affiliations:** 1 Department of Medicine and Aging Sciences, University “G. d’Annunzio” of Chieti-Pescara, 66013 Chieti, Italy; guagnano@unich.it (M.T.G.); chiara.dangelo@unich.it (C.D.); danielacaniglia@yahoo.it (D.C.); cellettieleonora@gmail.com (E.C.); sabatiniemanuela@gmail.com (E.S.); lorenza.speranza@unich.it (L.S.); marco.bucci@unich.it (M.B.); fcipollone@unich.it (F.C.); 2 Department of Pharmacy, University “G. d’Annunzio” of Chieti-Pescara, 66013 Chietii, Italy; pamela.digiovanni@unich.it; 3 YDA, Institute of Clinical Immunotherapy and Advanced Biological Treatments, 65121 Pescara, Italy

**Keywords:** rheumatoid arthritis, inflammation, pain, dietary regimen, meat- gluten- and lactose-exclusion diet, bioimpedance analysis, leptin

## Abstract

Introduction: Rheumatoid arthritis (RA) is a chronic systemic autoimmune disease affecting the synovial joints and causing severe disability. Environmental and lifestyle factors, including diet, have been proposed to play a role in the onset and severity of RA. Dietary manipulation may help to manage the symptoms of RA by lowering inflammation and potentially decreasing pain. Methods: In 40 patients with long-standing RA with stable symptoms and treated with conventional (c-) and biological (b-) disease modifying anti-rheumatic drugs (DMARDs), the effect of a 3-month diet avoiding meat, gluten, and lactose (and all dairy products; privative diet) was evaluated in comparison with a control balanced diet including those foods. Both diets were designed to reduce weight since all patients were overweight or obese. Patients were randomly assigned to one of the diets, and RA was clinically assessed at Time 0 (T0), through the Visual Analogue Scale (VAS), for pain, and the Disease Activity Score of 28 joints (DAS 28) for RA activity. Patients were also administered the Short Form Health survey (SF-36) and the Health Assessment Questionnaire (HAQ). At T0, a blood sample was collected for laboratory tests and adipokines measurements, and anthropometric measurements were compared. These evaluations were repeated at the end of the 3 months’ dietary regimens. Results: A significant decrease in VAS and the improvement of the overall state of physical and mental health, assessed through SF-36, was observed in patients assigned to the privative diet. Both dietary regimens resulted in the improvement of quality of life compared to baseline values; however, the change was significant only for the privative diet. With either diet, patients showed significant decreases in body weight and body mass index, with a reduction in waist and hips circumference and lower basal glucose and circulating leptin levels. A privative diet was also able to significantly reduce systolic (*p* = 0.003) and diastolic (*p* = 0.025) arterial pressure. The number of circulating leukocytes and neutrophils, and the level of hs-C-Reactive Protein also decreased after 3 months of the meat-, lactose-, and gluten-free diet. Conclusions: Our results suggest that a privative diet can result in a better control of inflammation in RA patients under stable optimized drug treatment.

## 1. Introduction

Rheumatoid arthritis (RA) is a chronic systemic inflammatory disease typically affecting the synovial joints, in which autoimmunity drives dysregulated proinflammatory cytokine secretion [1]. If not properly managed, RA leads to severe disability in most patients [2]. The increase in the incidence of autoimmune diseases observed in developed countries has been associated with a decrease in infectious load and dramatic changes in environmental and lifestyle factors, and particularly the diet, which affects the composition of the gut microbiota [3,4]. These factors, including the diet, have been postulated to play a role in the expression and severity of RA [5,6].

A decrease in dietary fibers and an increase in fat and sugar intake, which is typical in Western diets, contributes to gut microbial dysbiosis, leading to immune dysfunction [7,8,9,10,11]. The changes in the gastrointestinal microbiota are thought to influence the pathogenesis and progression of RA [12,13]. More importantly, they are amenable to correction through an appropriate diet, also through the restoration of microbial diversity [14,15].

Dietary manipulations have been used to manage the symptoms of RA [16,17,18], increasing antioxidant levels and altering lipid profiles, also by promoting a complex structure, such as the Mediterranean diet [19,20], and potentially modifying the intestinal flora [5,17,18]. The results of these studies showed that some foods exert clinically relevant inflammatory effects, and Mediterranean, vegetarian/vegan, and reduced-calorie/fasting diets, or nutritional supplements as fish oil, fibers and antioxidants, all result in some improvement in clinical activity in RA.

Nutritional studies reporting subjective beneficial effects [21,22] have been popularized and adopted by many patients. In this regard, consumption of red meat [23,24], dairy products or specifically lactose [25,26], and, more recently, gluten, although only small case series have been reported [27], have been indicted as possible causes of arthritis or of its exacerbations. 

In this study, we evaluated the effect of a diet deprived of meat, gluten, and lactose (excluding all dairy products and foods containing them) in patients with long-standing, well-controlled RA, in comparison with a control balanced diet including those items, followed for three months. The main endpoints of our study were the assessment of disease activity, pain perception, and modifications of quality of life in RA patients. Several inflammatory and anthropometric measurements were also recorded before and after the diet.

## 2. Materials and Methods

### 2.1. Patients Enrollment

A total of 40 patients with RA, fulfilling the classification criteria of the American College of Rheumatology (ACR)/European League Against Rheumatism (EULAR) [28], were recruited among those attending the Rheumatology Clinic of the hospital “SS. Annunziata” in Chieti. All were females with ages ranging between 31 and 72 years (mean age ± SEM: 52.23 ± 1.61), and all had been treated with the same optimized therapy for at least one year, consisting of a combination of biological (b-) and conventional (c-) Disease Modifying Antirheumatic drugs (DMARD) (*n* = 7), or monotherapy with a biological drug (*n* = 22), or c-DMARDs only (*n* = 11), showing stable disease activity, assessed with the scales described below. 

Comorbidities (diabetes, dyslipidemia, celiac disease), treatment with medium to high doses of corticosteroids (i.e., above 7.5 mg/day of prednisone equivalent), and current or previous dietary regimens with avoidance of meat, gluten, or milk represented exclusion criteria. The dietary habits of the patients were assessed by interview, showing a substantial adherence to the Mediterranean diet, which is prevalent in the region of the study, and remained unchanged in the previous years. However, individual consumption of meat, gluten, or milk was not quantified since this was not the purpose of the study. 

Moreover, patients were asked about physical activity and important lifestyle changes (significant increase in physical activity, job change, etc.). All patients reported low/moderate physical activity (e.g., casual walk, bike riding, jogging, or swimming in the pool) and no changes in lifestyle occurred in the previous year. Low-dose steroid therapy (less than 7.5 mg Prednisone equivalent/day) and occasional use of Nonsteroidal Anti-inflammatory Drugs (NSAIDs) or analgesics were allowed throughout the study. 

All the participants gave informed consent for study enrollment, as required from the local Ethics Committee for Biomedical Research which approved the study (Committee project no. 10/20). The investigations adhered to the Declaration of Helsinki, revised in 2013. 

### 2.2. Study Design and Dietary Regimens Protocol

Enrolled patients were randomly assigned to one of the two experimental groups and asked to follow a diet excluding meat, gluten and lactose (group A) or a balanced diet (group B). Groups were similar in age (mean ± SEM group A: 50.60 ± 2.24 years; mean ± SEM group B: 54.10 ± 2.09 years; *p* = 0.269) and main anthropometric characteristics.

Diets were designed by expert physicians at the Obesity Center of the “SS. Annunziata” hospital in Chieti; both diets gave an intake of about 1500 Kilocalories (Kcal)/day (as part of the treatment, since all patients were overweight/obese), and were optimized according to current guidelines for balanced composition in macronutrients [29], daily intake of cholesterol (<300 mg/die), saturated fatty acids (<10% of total energy intake) [30], oligosaccharides (<15% of total energy intake), and dietary fiber (25–30 g/die) [31]. Moreover, for both types of diet, the total protein intake was 50% from animal and 50% from vegetable proteins. 

The macronutrient composition of the meat-, gluten-, and lactose-deprived diet was as follows: 56% of total Kcal from carbohydrates, 16% from proteins, and 28% from fat. The main contributors to protein intake were fish (50.1%) for animal protein and flour products (16.1%), legumes (19.3%), and fruit and vegetables (14.5%) for vegetable protein. The macronutrient composition of the balanced diet was as follows: 56% carbohydrates, 17% proteins, and 27% fats. The main contributors to protein intake were poultry meat (28.8%) such as chicken and turkey and milk (products) (7.1%), for animal protein, and flour products (31.1%), legumes (15.7%), fruit and vegetables (17.3%) for vegetable protein. These data and the detailed description of the diets are reported in Supplemental Tables S1–S3. Red meat was absent in both diets, and all dairy products were eliminated in diet A. The diets were to be adhered to strictly every day, and detailed items allowed are listed in the supplemental tables.

In addition to the diets, advice for a correct lifestyle was provided. In particular: drinking at least 2 L/day of water/liquids, and walking at least 30 min/day, or 1 h in the gym 1–2 times/week, and general advice: do not smoke, do not drink alcohol, sleep regularly respecting the normal sleep/wake rhythm

### 2.3. Disease Monitoring

At the time of recruitment, Time 0 (T0), all patients underwent clinical evaluation of RA through an objective physical articular examination, also used to calculate the Disease Activity Score of 28 joints (DAS 28) (see below). The Visual Analogue Scale (VAS) was used for pain assessment, asking the patient to locate on a 100 mm line the point that best identified the intensity of pain in the previous week (where 0 represents no pain and 100 mm represents the maximal pain perceived) [32]. Patients were also asked how the disease condition affected their quality of life by administration of the Short Form Health survey (SF-36) [33] and the Health Assessment Questionnaire (HAQ) [34]. The activity of RA was evaluated by the 4-parameter DAS 28 using ESR [35].

All the above-described measures were repeated after 3 months of diet (T1) in both groups A and B. At the follow-up visit at T1, patients were inquired about adherence to the prescribed diets, and this was total for those who completed the 3 months of the study.

### 2.4. Laboratory Data and Anthropometric Measurements 

Patients underwent blood sample collection at T0 by venipuncture, and a complete blood count, and clinical chemistry tests were performed, including Oral Glucose Tolerance Test (OGTT), Homeostasis Model Assessment (HOMA) index, insulin level, serum lipid profile, Erythrocyte Sedimentation Rate (ESR), high-sensitivity C-Reactive Protein (hs-CRP), transaminase levels, total proteins, albumin, and transferrin. Serum aliquots, obtained after blood clotting and centrifugation, were stored at −80 °C, until assayed for circulating cytokines and adipokines evaluation. Anthropometric measurements, patients’ height, weight, and calculation of the Body Mass Index (BMI) (kg/m^2^), arterial blood pressure, and Bioimpedance Analysis (BIA) [36] were also recorded. BIA parameters were: muscle mass, fat mass, bone mass, water, and basal metabolism. All the above-described evaluations obtained at T0 were repeated after 3 months (T1) of the deprived (group A) or balanced (group B) diet.

### 2.5. Detection of Adipokines and Cytokines

Selected cytokines and adipokines were measured using the specific Quantikine^®^ ELISA kits (Human Leptin Immunoassay, Human Adiponectin Immunoassay, Human TNFα Immunoassay, Human IL-10 Immunoassay, and Human INFγ Immunoassay) purchased from R&D Systems (Minneapolis, MN, USA). All ELISA assays were performed on serum samples collected at T0 and T1, in the same batch, following the manufacturer’s instructions.

### 2.6. Statistical Analysis

Qualitative variables were reported as frequency and percentage, and quantitative variables as median and interquartile range (IQ). The chi-square test was used to compare proportions. Nonparametric statistics was used to compare the subjects at T0 and after the dietary intervention (Wilcoxon matched-pairs signed-rank test), and to compare the patients randomly assigned to group A or B at T0 and T1 (Mann–Whitney U-test). Spearman’s rank correlation coefficient was calculated to assess the relationship between the variables changes at T1 vs. T0. The threshold of statistical significance was set at *p* = 0.05. Data analysis was performed on GraphPad Prism 6 Software, version 6.01, 2012.

## 3. Results

### 3.1. Patients’ Adherence

A total of 12 patients withdrew from the study before T1, and this left 15 cases in group A and 13 in group B who completed the 3 months of the study without departure from the prescribed diet. Causes of poor compliance were difficulties to adhere to diet(s), also due to family reasons, in 9 patients (4 in group A and 5 in group B), and nonspecific gastrointestinal complaints (bloating, nausea) in 3 patients (1 in group A and 2 in group B). None of the patients reported worsening of symptoms or a flare of RA as reasons for leaving the trial.

### 3.2. VAS Score

**Figure 1:**
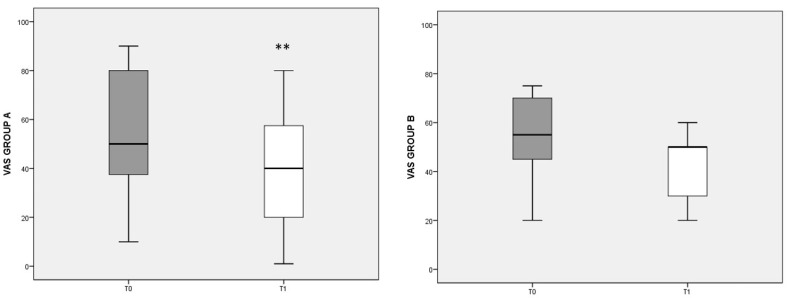
VAS score referred to different dietary regimens. Box plots represent the median and interquartile range of VAS scores in groups A and B. *p*-value derived from Wilcoxon matched-pairs signed rank test; ** *p* = 0.003 T1 VAS vs. T0 VAS.

To evaluate pain perception and its changes with dietary regimen, the VAS score was used (Figure 1). In group A, a significant decrease in VAS was found after the diet (T0 median: 50, IQ: 35–80; T1 median: 40, IQ: 10–60; *p* = 0.003). In group B, with the control diet, the VAS score also decreased, but not significantly.

### 3.3. DAS 28 Score

**Figure 2:**
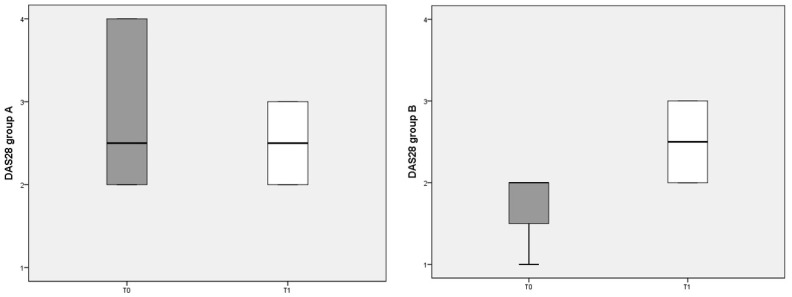
DAS 28 scores referred to different dietary regimens. Box plots represent the median and interquartile range of DAS 28 index in the two groups (A and B diets).

The composite clinimetric index DAS 28 was used to evaluate changes in RA activity after 3 months of diet (Figure 2). The DAS 28 score was not significantly different before and after either diet. 

### 3.4. SF-36 Score

**Figure 3:**
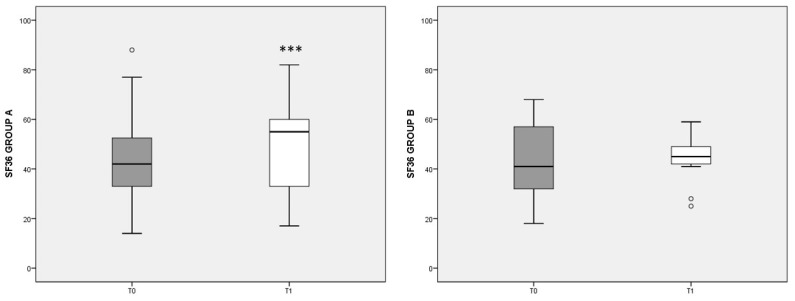
Total SF-36 score referred to different dietary regimens. Box plots graphically represent the median and interquartile range of SF-36 total score in groups A and B. *p*-value derived from Wilcoxon matched-pairs signed rank test; *** *p* < 0.001 T1 SF-36 vs. T0 SF-36.

Figure 3 summarizes the eight scales included in the SF-36 questionnaire, showing the overall state of physical and mental health in patients at the beginning (T0) and after 3 months’ diet (T1). Note that higher values indicate a better health status. In group A, the T1 score (median: 59, IQ: 46–74) was significantly increased (*p* < 0.001) with respect to T0 values (median: 42, IQ: 30–54), whereas in group B no significant change was detected. The differences at T1 tended to favor the outcome of diet A (*p* = 0.056).

When the eight items of the SF-36 questionnaire were considered apart, in group A we observed a significant increase in the state of health (SH), the vitality (V), the social activities (SA), and the role limitations due to the emotional state (RE) scores. In group A, the increase in the V score was inversely correlated with pain reduction, assessed by the VAS (rho = −0.546, *p* = 0.027). In group B, the increase in the eight items of SF-36, with respect to T0, were smaller and did not reach statistical significance, with SH, V, and mental health (MH) scores showing no variation with respect to T0 (data not shown). 

### 3.5. HAQ Score

**Figure 4:**
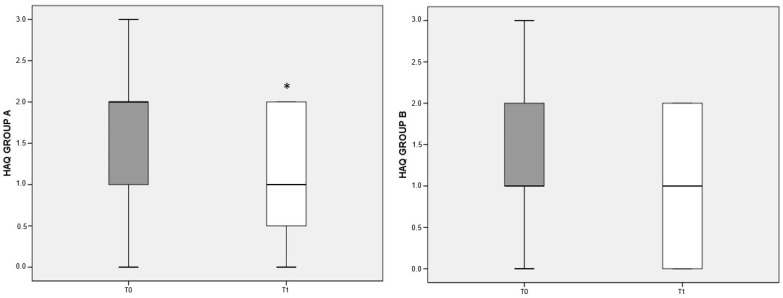
HAQ variation referred to different dietary regimens. Box plots graphically depict the median and interquartile range of HAQ score in groups A and B. *p*-value derived from Wilcoxon matched-pairs signed rank test; * *p* < 0.05 T1 HAQ vs. T0 HAQ.

The items on the patient’s physical, psychological, and social dimensions in the HAQ test are organized so that a lower score indicates a better quality of life. Both the deprived and the balanced diet were associated with improved quality of life in RA patients compared to baseline; however, the difference reached significance (*p* < 0.05) only for patients belonging to group A (Figure 4). 

### 3.6. Anthropometric Measures

The anthropometric measures are reported in Table 1. Patients randomly assigned to group A or B at T0 were not significantly different for all variables considered (penultimate column of Table 1). After 3 months of the two dietary regimes, patients showed a significant decrease in body weight and BMI (Table 1). All patients were overweight/obese at T0. In particular, eight (50%) females in group A and eight (50%) in group B were overweight and five (41.7%) females in group A and seven (58.3%) in group B were obese, but this distribution is not statistically significant (*p* = 0.662) (data not shown). Both dietary regimes were also significantly effective in reducing waist and hips circumference. Interestingly, in group A, a significant reduction in systolic (SYS) (*p* = 0.003) and diastolic (DIA) (*p* = 0.025) arterial pressure was achieved, but this did not occur in group B. Among the BIA parameters, no change in muscle mass and basal metabolic rate was observed after diet in either group, whereas fat mass was significantly reduced in both. The last column of Table shows the statistical differences between variables in group A and B at time T1. It is possible to note that there are statistically significant differences between the two diets in muscle mass and water, which have higher values in group A, and in systolic blood pressure, which has a lower median value in group A.

In group A, the lower weight, BMI, and fat mass, were significantly correlated with increased V scores in the SF-36 questionnaire (rho = −0.527, *p* = 0.038; rho = −0.546, *p* = 0.045; rho = −0.510, *p* = 0.033, respectively). Moreover, statistically significant correlations between weight loss, reduction in hips circumference, and the RP score (used to assess role limitations due to physical health; rho = −0.610, *p* = 0.009 and rho = −0.576, *p* = 0.013, respectively) were found. In group B, after 3 months of the control balanced diet, a significant decrease in fat mass and water content have been measured (Table 1).

The improvement in the HAQ score observed in both groups can be traced back to the moderate calorie restriction to which patients were subjected, such that the produced weight reduction significantly correlated with HAQ in group B (rho = 0.586, *p* = 0.044).

### 3.7. Laboratory Parameters

The metabolic, hematological, and biochemical parameters analyzed are reported in Table 2. Patients randomly assigned to groups A or B at T0 did not differ significantly (penultimate column of Table 2).

Basal glucose levels in patients after 3 months of either dietary regimen showed a statistically significant decrease (Table 2). The deprived diet was also effective in significantly reducing the HOMA index [37] in group A. No significant changes in fasting insulin levels, blood lipid profile, total proteins, or albumin were recorded (Table 2). No differences were observed between the groups at T1 (see last column).

The number of circulating leukocytes and neutrophils, and the level of hs-CRP were also significantly decreased in group A, together with a significant reduction in transferrin (*p* = 0.013). The decline in leukocyte numbers was inversely correlated with an increase in the physical pain score (PP) item in the SF-36 test (rho = −0.579, *p* = 0.026). 

### 3.8. Adipokines and Cytokines Measurements

**Figure 5:**
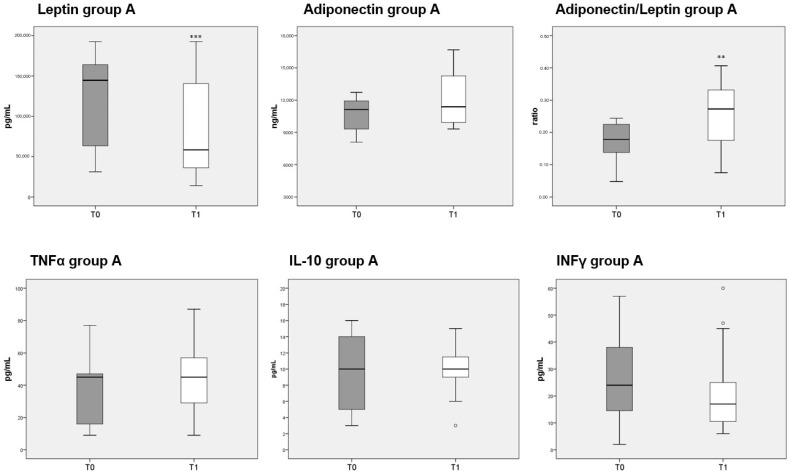
Box plots indicate the median and interquartile range of adipokines and cytokines measured in sera of patients belonging to group A. *p*-values derived from Wilcoxon matched-pairs signed rank test; ** *p* < 0.01, *** *p* < 0.001, T1 vs. T0.

**Figure 6:**
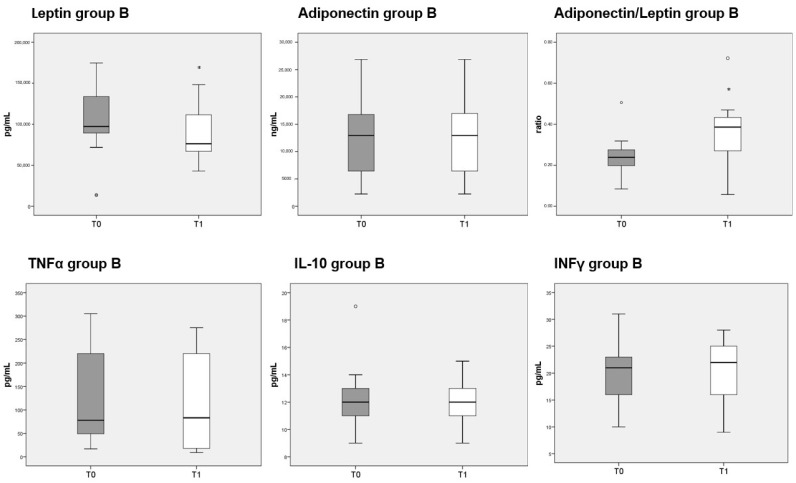
Box plots show the median and interquartile range of adipokines and cytokines measured in sera of patients belonging to group B. *p*-values derived from Wilcoxon matched-pairs signed rank test; * *p* < 0.05, T1 vs. T0.

Circulating levels of leptin were significantly decreased after 3 months of diet in both groups A and B, whereas adiponectin was unaffected. The reduction in leptin caused a significant increase in the adiponectin/leptin ratio in both group A (Figure 5, top third panel) and B (Figure 6, top third panel). The serum levels of the three cytokines measured (TNFα, IL-10 and INFγ) were not significantly different before or after either diet.

## 4. Discussion

Our study enrolled RA patients with a long-standing, well-controlled condition under stable pharmacological treatment, and subjected them to a trial diet with the exclusion of three food items (meat, gluten, and lactose) that have been suspected of aggravating the symptoms and worsening the disease course, in comparison with a control balanced diet including those foods. The results demonstrate that both diets, despite not affecting the disease activity, measured by the 4-parameter DAS28, had a tendency to reduce the arthritic pain perception and to improve the quality of life of the patients. Moreover, metabolic and inflammatory parameters showed a trend to normalize, and this, together with a significant lowering of systolic blood pressure might impact the prognosis and the long-term cardiovascular risk of RA [38,39]. 

We hypothesized that the two diets may be influencing disease perception and RA burden in several ways, through a range of mechanisms, among them normalizing the adiponectin-to-leptin ratio which is a sensitive index of the inflammatory status [40], adipose tissue dysfunction, and reducing cardiometabolic risk [41]. The decrease in inflammation was reflected by the reduction in hs-CRP, but not in ESR and TNF-α. These modifications may explain the lack of effect on RA activity, measured by DAS-28, and the changes in glucidic homeostasis (HOMA index), as well as mental and physical health [42,43].

Both diets had significant impact on weight reduction, thus lowering the BMI, and BIA revealed a significant decrease in fat mass, with lesser changes for other measurements. This lowering of weight was indeed one of the therapeutic goals of the diets, which were designed to have a low calorie content, considering that all patients selected were overweight/obese, despite following a habitual Mediterranean diet. However, only the exclusion diet induced significant changes in adipokines, CRP, and glucidic control compared to T0. Moreover, it achieved a significant decrease in both SYS and DIA blood pressure, and the systolic was reduced also when compared with T1 levels of diet B. Therefore, we cannot ascribe both diets’ results to the simple loss of extra weight and fat. In our RA patients, 3 months of a deprived diet was able to significantly reduce total leukocytes and neutrophils number, and CRP and transferrin levels as well. Comparing the two diets at time 1, we found statistically significant differences in the median value of SYS blood pressure (lower in the deprived diet group) and in median values of muscle mass and water (higher in the deprived diet group).

Several types of diet or nutritional supplements in RA have received increased attention in recent years, and dietary intervention studies in RA have been published [20,44,45,46,47,48]. A 3-month Mediterranean-based diet in RA patients ensured clinical benefits, with a significant reduction in DAS28 and improved quality of life assessed by HAQ and SF-36 compared to RA control patients on their usual diets [49]. In that study, only recommendations were given, and the diets differed under all aspects. However, there is limited but univocal evidence to suggest that the Mediterranean diet is beneficial in the prevention and treatment of pathological conditions, including RA [5,18,50]. The protective effect of the Mediterranean diet on RA disease activity may be due to changes in the gut microbiota of the patients as shown in the study Prevention with Mediterranean Diet-(PREDIMED): [51]. The Mediterranean diet shows many similarities to the dietary schemes adopted in our study, but we failed to observe a reduction in disease activity. We must also point out that all our patients, though not assessed with precision, habitually followed a Mediterranean-style diet before entering the study. 

Enrolled patients did not show significant differences at the recruitment time, also considering the different pharmacological treatment received. The low-calorie content was equal for the two diets, and animal protein was provided in both, thus ruling out other possible explanations based on vegetarian diets or fasting. Among rheumatic patients who switched from an omnivorous to a vegetarian diet, an attenuation of disease activity has been reported [48], and fasting, even for short duration, or fast mimicking eating methods have been shown to provide some advantageous outcomes to RA patients [52].

Some dietary components may affect chronic inflammatory diseases, although a large prospective study of 56,075 Danish middle-aged men and women failed to detect a higher risk of any inflammatory disease, or RA specifically, for low fiber or/and high meat intake [53]. Both diets in our study excluded red meat, and only chicken and turkey were allowed in diet B (control diet).

Obesity in RA has been found to be associated with higher DAS28, tender joint counts, inflammatory marker levels, patient global evaluation and pain scores, as well as physical function scores [54,55,56]. However, it is not associated with increased mortality [57]. Many of our patients were obese; their disease activity was kept under control by pharmacological treatment, and it did not change throughout the study, even after weight reduction. Altered body composition is very frequent in RA, and the majority may present with loss of lean mass, the so-called rheumatoid cachexia. Only a non-significant reduction in muscle mass was observed for both diets in our study; however, this resulted in a significant difference at T1, with lower mass in group B. Leptin levels, which are raised in obese patients, are markedly increased in RA and related to general inflammatory status and disease activity [58]. In our study, both diets showed a tendency to lower the leptin levels, resulting in an increase in the adiponectin/leptin ratio, significant only in the exclusion diet.

Diet has a central role in increasing inflammatory disease risk and progression. Several nutrients may have protective activity for RA symptoms, whereas others may have a harmful role. The use of fish-rich diets, among them the Mediterranean diet, or fish-oil supplementation in long-standing RA, have proven beneficial in meta-analyses [59], so the effect of substituting fish for meat as a source of animal protein in our study may be questioned. Different types of fish were allowed though, with widely variable content in omega-3 and other components, so the question is purely speculative and difficult to answer. 

This study has some strengths and several limitations. The small number of patients enrolled calls for the intention to be a pilot study, and the high number of dropouts, mainly due to the problems of maintaining strict diets for a long time (i.e., not allowing for holidays) reduced the final study group to 28 RA patients. The patients were all female and overweight or obese, so they might not be representative of all cases. Individual histories might have differed too, as did ongoing and past treatments, as well as their microbiota; future studies should take all these variables into account. No intermediate time point was used to check the study course for its three months’ duration. Aside from these limitations, our study has some strengths: the prescription of clearly identified diets, and their balanced composition of macronutrients. Both groups had to adhere to a strict diet, and the therapeutic goal of the diet was to reduce excess weight. Lastly, the diet had to be continued for a reasonable time, 3 months, in order to observe more stable changes.

Although attenuating effects on RA pathology were observed in our and others’ dietary intervention studies, none of them have been successfully implemented in clinics so far. Maybe the reasons are due to the poorly characterized mechanisms underlying the improvement in RA patients, or the availability of ever more effective drugs that make dietary alternatives less appealing. Moreover, all dietary interventions require changes to the patients’ daily lifestyle and eating behaviors, and that requires commitment, endurance, persuasion, and time. 

## 5. Conclusions

The results obtained appear to confirm that exclusion of three common food items, all reported as suspected of worsening of symptoms in RA, with the inclusion of fish, may carry additional health benefits to overweight patients compared with those obtained by a standard weight-reduction program (diet B). The immunological correlates of RA were not sensitive to either diet, but measures of general physical and mental state, pain perception and quality of life showed a tendency to improve with both. The better preservation of muscle mass and the lower systolic blood pressure achieved with the exclusion diet A shows that weight loss in our patients was not the only explanation, since both diets had this result. The aim of preventing complications of RA might be better achieved by the exclusion diet, so a diet prescription should be included in therapeutic plans for RA.

## Figures and Tables

**Table 1:** Anthropometric measures of recruited patients in groups A and B.

	Group A (*n* = 15)		*p*-Value ^a^	Group B (*n* = 13)		*p*-Value ^a^	*p*-Value ^b^	*p*-Value ^c^
	T0	T1		T0	T1			
Weight (Kg)	73 (68–87)	72 (64–82)	**<0.001**	72 (65–80)	67 (64–75.5)	**0.003**	0.270	0.420
BMI (kg/m2)	29 (26–36)	27 (25–33)	**<0.001**	29 (25–33.5)	26 (24–31.5)	**0.012**	0.355	0.677
BIA:								
Muscle mass (Kg)	44 (41–45)	43 (41–45)	0.188	41 (39.5–42.5)	40 (38.5–42)	0.070	0.058	**0.030**
Fat mass (Kg)	26 (23–39)	25 (20–35)	**<0.001**	28 (22.5–35)	25 (20–32)	**0.015**	0.533	0.926
Bone mass (Kg)	2 (2–2)	2 (2–2)	0.564	2 (2–3)	2 (1–2)	0.157	0.097	0.636
Water (Kg)	33 (31–34)	32 (30–34)	0.256	31 (29–33)	29 (28.5–31)	**0.028**	0.138	**0.045**
Basal metabolism (Kcal)	1375 (1305–1456)	1368 (1294–1445)	0.379	1308 (1258–1380)	1293 (1241–1364)	0.084	0.093	0.096
Waist circumference (cm)	101 (97–105)	93 (82–106)	**<0.001**	101 (97–104)	95 (92–98)	**0.005**	0.890	0.948
Hips circumference (cm)	110 (102–119)	104 (98–110)	**<0.001**	111 (108–114)	107 (104–110)	**0.013**	0.945	0.746
Waist Hips Ratio (WHR)	0.92 (0.89–0.94)	0.91 (0.85–0.94)	0.543	0.91 (0.89–0.93)	0.88 (0.86–0.91)	0.059	1.000	0.504
SYS (mmHg)	131 (127–136)	120 (116–124)	**0.003**	125 (122–128)	127 (123–130)	0.103	0.856	**0.036**
DIA (mmHg)	80 (79–84)	74 (71–76)	**0.025**	80 (70–80)	75 (70–80)	0.253	0.316	0.964

BMI: Body Mass Index, BIA: body impedance analysis, SYS: systolic arterial pressure, DIA: diastolic arterial pressure. Data are expressed as median and interquartile range (IQ). ^a^ *p*-value derived from Wilcoxon matched-pairs signed rank test T1 (after 3 months’ diet) vs. T0 (enrollment and start of the diets). ^b^ *p*-value derived from Mann–Whitney test T0 group A vs. T0 group B. ^c^ value derived from Mann–Whitney test T1 group A vs. T1 group B. The significant differences are marked in bold.

**Table 2:** Laboratory data of recruited patients in group A and B.

	Group A (*n* = 15)		*p*-Value ^a^	Group B (*n* = 13)		*p*-Value ^a^	*p*-Value ^b^	*p*-Value ^c^
	T0	T1		T0	T1			
OGTT:								
Basal (mg/dL)	93 (81–98)	85 (80–90)	**<0.001**	90 (85–94.5)	85 (80–90)	**0.003**	0.419	0.888
After 120 min (mg/dL)	129 (112–139)		-	131 (109–145.5)		-	0.872	-
Insulin (μUL/mL)	11 (6–18)	9 (6–11)	0.070	8 (6–10)	9 (5–11.5)	0.632	0.343	0.817
HOMA index	3 (1–4)	2 (1–2)	**0.044**	2 (1–2)	2 (1–2.5)	0.705	0.201	0.939
Total Cholesterol (mg/dL)	202 (177–226)	196 (186–208)	0.426	203 (181.5–229)	213 (174.5–237)	0.456	0.712	0.300
HDL-Cholesterol (mg/dL)	60 (46–77)	60 (47–76)	0.858	58 (50.5–67.5)	60 (54–67.5)	0.452	0.963	0.945
Triglycerides (mg/dL)	137 (64–195)	102 (72–163)	0.064	102 (76–171)	101 (67–136.5)	0.147	0.747	0.565
Leukocytes (*n* × 10^3^/μL)	8 (6–9)	6 (6–8)	**0.003**	7 (6–8)	6 (5–7.5)	0.114	0.270	0.419
Neutrophils (*n* × 10^3^/μL)	5 (3–5)	3 (3–4)	**0.006**	4 (3–5)	4 (2.5–4)	0.103	0.408	0.566
Lymphocytes (*n* × 10^3^/μL)	3 (2–3)	2 (2–3)	0.180	3 (2–3)	2 (2–3)	0.256	0.592	0.662
Hemoglobin (g/dL)	13 (13–14)	13 (13–14)	0.655	14 (13–14)	13 (13–14)	0.248	0.074	0.507
Platelets (*n* × 10^3^/μL)	279 (234–323)	269 (223–303)	0.342	283 (241–321.5)	267 (214.5–329.5)	0.184	0.612	0.945
ESR (mm/h)	21 (18–25)	21 (18–24)	0.735	16 (13–20.5)	22 (18–25)	0.069		0.885
Total Proteins (g/dL)	7 (7–8)	7 (7–8)	0.414	7 (7–8)	7 (7–7.5)	0.317	0.891	0.830
hs-CRP (mg/L)	0.7 (0.5–0.9)	0.5 (0.4–0.5)	**0.032**	0.6 (0.4–0.7)	0.5 (0.4–0.55)	0.306		0.960
Albumin (g/dL)	4 (4–4)	4 (4–5)	0.346	4 (4–4)	4 (4–5)	0.564	0.856	0.413
Transferrin (mg/dL)	280 (263–304)	266 (239–302)	**0.016**	292 (264–300.5)	284 (272–295)	0.126	0.549	0.596
GOT (U/L)	25 (21–44)	28 (21–33)	0.222	23 (19–28.5)	24 (20.5–28.5)	0.875	0.249	0.344
GPT (U/L)	38 (24–54)	34 (26–45)	0.875	25 (21–41.5)	28 (23.5–34)	0.727	0.254	0.128

OGTT: Oral Glucose Tolerance Test, HOMA index: Homeostasis Model Assessment, HDL: High density lipoprotein, ESR: Erythrocyte sedimentation rate, CRP: C-Reactive Protein, GOT: Glutamic-Ossalacetic Transaminase, GPT: Glutamic-Pyruvic Transaminase. Data are expressed as median and interquartile range (IQ). ^a^ *p*-value derived from Wilcoxon matched-pairs signed rank test T1 (after 3 months’ diet) vs. T0 (enrollment and start of the diets). ^b^ *p*-value derived from Mann–Whitney test T0 group A vs. T0 group B. ^c^ *p*-value derived from Mann–Whitney test T1 group A vs. T1 group B. The significant differences are marked in bold.

## Data Availability

The data that support the findings of this study are available from the corresponding author upon reasonable request.

## Supplementary Materials

The following are available online at https://www.mdpi.com/article/10.3390/nu13103535/s1, Table S1: Macronutrients (daily intake). Table S2: Privative diet (deprived of meat, gluten and lactose) (1500 Kcalories). Table S3: Balanced diet (1500 Kcalories).

## Abbreviations

RA: Rheumatoid arthritis; c-, b-DMARDs: conventional-, biological-disease modifying anti-rheumatic drugs; NSAIDs: Nonsteroidal Anti-inflammatory Drugs; DAS 28: Disease Activity Score of 28 joints; VAS: Visual Analogue Scale; SF-36: Short Form Health survey; HAQ: Health Assessment Questionnaire; OGTT: Oral Glucose Tolerance Test; HOMA: Homeostasis Model Assessment index; ESR: Erythrocyte Sedimentation Rate; hs-CRP: high sensivity-C-Reactive Protein; BMI: body mass index; BIA: Bioimpedance Analysis; SYS: systolic arterial pressure; DIA: diastolic arterial pressure.
