# Supplementary material for: Improvement of Inflammation and Pain after Three Months’ Exclusion Diet in Rheumatoid Arthritis Patients
Source: Nutrients. 2021 Oct 9;13(10):3535. doi: 10.3390/nu13103535 (PMC8539601; doi:10.3390/nu13103535)
Supplement: Supplementary file 1 [file nutrients-13-03535-s001.zip › nutrients-1374891-supplementary.pdf]

Supplemental Table S1: Macronutrients (daily intake)

	PRIVATIVE DIET	BALANCED DIET
**CARBOHYDRATES**	**56%**	**56%**
**TOTAL PROTEINS**	**16%**	**17%**
Fish proteins	50,1 %	---
Meat proteins	---	28,8%
Milk product proteins	---	7,1%
Animal proteins & Flour products	16,1%	31,1%
Legumes proteins	19,3%	15,7%
Fruit & Vegetable proteins	14,5%	17,3%
**LIPIDS**	**28%**	**27%**

Supplemental Table S2: Privative diet (deprived of meat, gluten and lactose) (1500 Kcalories)

**Breakfast:**
Thé 200 g<br>Biscuits - (Bi-Aglut) 40 g
**Morning snack:**
Bananas 150 g<br>Dark chocolate 10 g
**Lunch:**
Paste - (Glutafin) 70 g<br>Dried legumes 40 g<br>Vegetables 200 g<br>Extra virgin olive oil 10 g
**Snack:**
Pears 200 g
**Dinner:**
Fresh cod or hake 180 g (or Sea bream fillets 110 g) (or Plaice or flounder 160 g) (or Fresh salmon 70 g) (or Sole 150 g) (or Sea bass 160 g)<br>Mixed salad 100 g<br>Carrots 100 g<br>Gluten-free bread 100 g<br>Extra virgin olive oil 20 g

Supplemental Table S3 – Balanced diet (1500 Kcalories)

**Breakfast:**
Partially skimmed pasteurized cow's milk 150 g<br>Wholemeal biscuits 40 g

**Morning snack:**
Oranges 150 g

**Lunch:**
Semolina pasta 80 g<br>Canned borlotti beans 120 g<br>Vegetables 100 g<br>Extra virgin olive oil 10 g

**Snack:**
Apples 150 g<br>Dark chocolate 10 g

**Dinner:**
Whole skinless chicken 120g (or chicken breast 130g) (or turkey breast 120g) (or turkey leg 110g)<br>Vegetables 200 g<br>Wholemeal bread 100 g<br>Extra virgin olive oil 15 g
